# Supplementary figures and images for: Landscape Dynamics and Ecological Risk Assessment of Cold Temperate Forest Moose Habitat in the Great Khingan Mountains, China
Source: Biology (Basel). 2023 Aug 11;12(8):1122. doi: 10.3390/biology12081122 (PMC10451888; doi:10.3390/biology12081122)

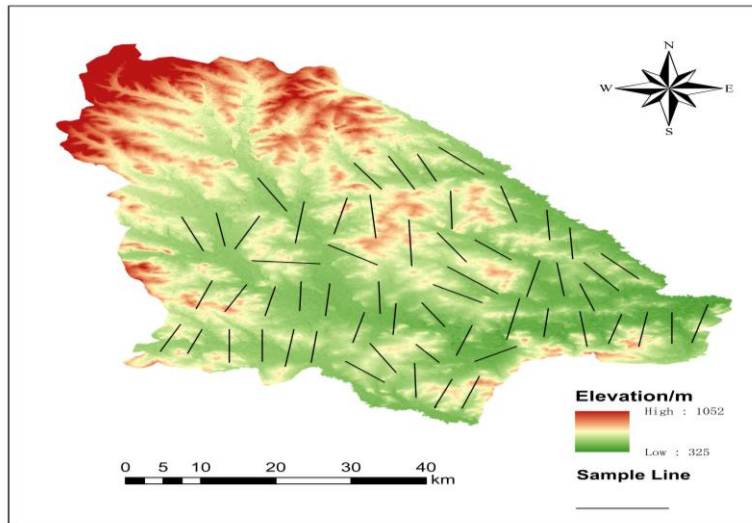

**Figure S1.** Sample line transect design diagram of study area.

Supplement: Supplementary file 1 [file biology-12-01122-s001.zip › biology-2516993-supplementary.pdf]
